# Vaccine coverage and determinants of incomplete vaccination in children aged 12–23 months in Dschang, West Region, Cameroon: a cross-sectional survey during a polio outbreak

**DOI:** 10.1186/s12889-015-2000-2

**Published:** 2015-07-10

**Authors:** Gianluca Russo, Alessandro Miglietta, Patrizio Pezzotti, Rodrigue Mabvouna Biguioh, Georges Bouting Mayaka, Martin Sanou Sobze, Paola Stefanelli, Vincenzo Vullo, Giovanni Rezza

**Affiliations:** Department of Public Health and Infectious Diseases, Faculty of Medicine and Pharmacy, “Sapienza” University of Rome, Piazzale Aldo Moro 5, 00185 Rome, Italy; Department of Infectious, Parasitic and Immune-mediated Diseases, Istituto Superiore di Sanità, Rome, Italy; Department of Biomedical Sciences, Faculty of Sciences, University of Dschang, Dschang, Cameroon; Dschang District Hospital, Dschang Health District, Dschang, Cameroon

**Keywords:** Vaccination coverage, Survey, Determinants of immunization, Children, Cameroon

## Abstract

**Background:**

Inadequate immunization coverage with increased risk of vaccine preventable diseases outbreaks remains a problem in Africa. Moreover, different factors contribute to incomplete vaccination status. This study was performed in Dschang (West Region, Cameroon), during the polio outbreak occurred in October 2013, in order to estimate the immunization coverage among children aged 12–23 months, to identify determinants for incomplete vaccination status and to assess the risk of poliovirus spread in the study population.

**Methods:**

A cross-sectional household survey was conducted in November-December 2013, using the WHO two-stage sampling design. An interviewer-administered questionnaire was used to obtain information from consenting parents of children aged 12–23 months. Vaccination coverage was assessed by vaccination card and parents’ recall. Chi-square test and multilevel logistic regression model were used to identify the determinants of incomplete immunization status. Statistical significance was set at *p* < 0.05.

**Results:**

Overall, 3248 households were visited and 502 children were enrolled. Complete immunization coverage was 85.9 % and 84.5 %, according to card plus parents’ recall and card only, respectively. All children had received at least one routine vaccination, the OPV-3 (Oral Polio Vaccine) coverage was >90 %, and 73.4 % children completed the recommended vaccinations before 1-year of age. In the final multilevel logistic regression model, factors significantly associated with incomplete immunization status were: retention of immunization card (AOR: 7.89; 95 % CI: 1.08–57.37), lower mothers’ utilization of antenatal care (ANC) services (AOR:1.25; 95 % CI: 1.07–63.75), being the ≥3^rd^ born child in the family (AOR: 425.4; 95 % CI: 9.6–18,808), younger mothers’ age (AOR: 49.55; 95 % CI: 1.59–1544), parents’ negative attitude towards immunization (AOR: 20.2; 95 % CI: 1.46–278.9), and poorer parents’ exposure to information on vaccination (AOR: 28.07; 95 % CI: 2.26–348.1). Longer distance from the vaccination centers was marginally significant (*p* = 0.05).

**Conclusion:**

Vaccination coverage was high; however, 1 out of 7 children was partially vaccinated, and 1 out of 4 did not complete timely the recommended vaccinations. In order to improve the immunization coverage, it is necessary to strengthen ANC services, and to improve parents’ information and attitude towards immunization, targeting younger parents and families living far away from vaccination centers, using appropriate communication strategies. Finally, the estimated OPV-3 coverage is reassuring in relation to the ongoing polio outbreak.

## Background

Despite the improvement in global immunization coverage, about 16 % of the worlds’ children had not completed the 3-dose diphtheria-tetanus-pertussis vaccine (DTP-3) series by 2013 [1]. The estimated 2013 global DTP-3 coverage among children aged <12 months, which is a key indicator of immunization program performance, was 75 % in the World Health Organization (WHO) African Region, and 84 % worldwide [1]. However, thanks also to the Expanded Program on Immunization (EPI), children immunization coverage in the WHO African Region is improving, even if still sub-optimal in many areas [2].

The EPI was introduced in Cameroon in 1976 [3]. According to the 2011 Cameroonian Demographic Health Survey (DHS) [4], the coverage of the nine EPI vaccines in children aged 12–23 months was 57 % at the national level, and 59.3 % in the West Region. In this region, wild poliovirus (WPV) type 1 was isolated from two acute flaccid paralysis (AFP) cases in October 2013 [5]. In 2013, other four cases due to circulating vaccine-derived poliovirus type 2 (cVPDPV-2) were reported also in the Far North Region of Cameroon. According to the Global Polio Eradication Initiative (GPEI), a total of nine cases of WPV-1 have been reported in Cameroon during the years 2013–2014 [6], representing the first polio-outbreak reported in the country since 2009.

Vaccine preventable diseases outbreaks are linked to inadequate levels of immunization coverage [5]. Several factors are associated with poor vaccination coverage in resources-limited countries. A multilevel analysis involving 24 African countries [7] showed that breaks in childhood vaccination are linked, at the contextual level, to high community illiteracy rates, high country fertility rates, and living in urban areas, while, at the individual level, they are linked to poorest households, uneducated parents, parents with no access to media and/or with low health seeking behaviours; the relative effect of the above factors may significantly vary according to the geographical area [7].

In Africa, a more detailed and comprehensive information at district level is necessary in order to develop and implement appropriate strategies for improving immunization coverage. In Cameroon, there are no studies, conducted at the district level, assessing factors associated with incomplete immunization. To fill this gap, we planned a community-based cross-sectional survey in the district of Dschang (West Region, Cameroon), a rural setting sited only 30 km far from the area affected by the polio outbreak [8]. The objectives of this study were to estimate the districts’ coverage of routine vaccination program, to identify risk factors for incomplete vaccination status among children 12–23 months old, and to assess the risk of WPV spread in the study population.

## Methods

### Study area

Dschang was selected as the study site because it is close to the outbreak area, and because of an established collaboration between the research team and local health authorities. The district of Dschang is located in the Menoua Department, West Region, Cameroon. According to local health authority, the 2012 districts’ estimated population was of 218,006 inhabitants (17 % under-five years old children), mostly belonging to the Bamiléké ethnic group and Christian religion. The local economy is based on agriculture and livestock. The district has 4 hospitals, 3 public medical centres, and 46 health centres that deliver EPI vaccines to the target population groups. The 2012 Dschang districts’ annual health report indicated a coverage of 90 % for all vaccines. During 2013, considering the increased risk of polio spread, Dschang district was involved in two national immunization days (NIDs) (October and December). Our study focused only on vaccinations received through the routine immunization program because, according to GPEI strategy [9], OPV doses provided during supplementary immunization activities (SIAs) are not registered on the vaccination card, but children are just marked on the finger.

### Study design and sampling

We conducted a cross-sectional household survey of children aged 12–23 months, between the 10^th^ of November and the 10^th^ of December 2013. Considering the unavailability of an exhaustive list of households and of children aged 12–23 months, a two-stage sampling design, using firstly the village/neighbourhood (clusters) and then the EPI random walk method, was performed [10]. At the first stage, the clusters were sampled with probability proportional to their size, according to the estimated population data per village provided by the health’ district authority. Assuming an expected vaccination coverage of 50 % (i.e., the percentage with the highest variability), an intracluster correlation coefficient of 0.04 (usually between 0.01 and 0.02 in human study surveys [11–15]), and a precision of ±5 %, a sample of 60 clusters of 9 children each, for a total sample size of 540 children aged 12–23 months, was initially planned. At the second stage, according to the EPI-style random walk [10], the sampling started from the geographic centre of the cluster. The direction of data collection was randomly chosen, using the spinning bottle method, and every house in that direction was selected for the survey, following the principle of the next nearest household. Children were recruited consecutively until the planned cluster sample size was achieved.

### Data collection

The Cameroonian immunization schedule at the time of the study is summarized in Table 1. After the identification of a child aged 12-to-23 months from the household selected through house-to-house visits, the presence of child’ immunization card was assessed. In case of two or more 12–23 months old children were found in the same house, the youngest child was selected [10]. A structured interviewer-administered questionnaire was used to obtain information from consenting parents. All interviewers were trained on the questionnaire use and EPI-style random walk. The acceptability and the logical structure of the questionnaire were checked on the field, during pre-testing. The questionnaire was constructed from a review of the available literature on immunization surveys in developing countries [10, 16–24], with the aim to investigate variables related to: parental, household and child characteristics; antenatal/postnatal care; parental perception, knowledge and attitude on immunization; parental exposure to information on immunization; child vaccination history; reasons for non-vaccination. Data on child vaccination (doses, timing and type of vaccines received) were collected from vaccination cards and, if unavailable, by parents’ interview only, as suggested by WHO [10]. Information on other variables was collected by parents’ interview only.

**Table 1:** Cameroonian vaccine schedule^a^

Vaccine	Schedule
BCG (Bacille Camlmette Guérin)	Birth
OPV (Oral Polio Vaccine)	Birth; 6, 10, 14 weeks
DTwPHibHepB (Diphtheria and Tetanus toxoid with whole cell Pertussis, *Haemophilus Influenzae* type B and Hepatitis B)	6,10,14 weeks
Measles	9 months
YF (Yellow Fever)	9 months
PCV (Pneumococcal conjugate vaccine)^b^	6,10,14 weeks^b^

^a^Schedule at the time of the performed survey (November 2013)

^b^At the time of the survey the oldest enrolled children were born in December 2011, just few months after the decision of introducing PCV in Cameroon. We decided not to address PCV coverage because the oldest children could not have had access to PCV vaccine supply

### Data analysis

After reviewing for completeness and accuracy, all data from eligible children were included in a database. Double data entry was performed and validated. The parents’ vaccination knowledge, their attitudes towards immunizations and their opinion on Dschang immunization services were assessed through a 5-points scale (1 point per each correct response) closed-ended questions (5 for each area) and classified respectively as good or poor knowledge, negative or positive attitudes, and bad or good opinion. A minimum of 3-points score was established as cut-off. Parents’ exposure to information about immunization was assessed through 4 weighed open questions and classified as present or absent. The socio-economic household status was classified in tertiles, through an algorithm that considered the type of family (two or single-parent), and the parents’ occupation. These scoring systems were constructed from a review of the available literature on immunization surveys in developing countries [16–24]. Children vaccination status definitions are summarized in Table 2. The risk of WPV spread was assessed measuring OPV3 coverage in accordance with GPEI [25]. The dropout rate was defined as the percentage of children who received the 1^st^ but not the 3^rd^ dose of vaccines, or who received the first (BCG, Bacille Calmette Guérin) but not the latest (measles or yellow fever) vaccine included in the vaccine schedule. The dropout rates (by card and by card plus parents’ recall) were evaluated according to WHO calculation method: [(first dose – last dose)/first dose] ×100 [26].

**Table 2:** Operational definitions of child vaccine status

Vaccine status	Definition
Fully vaccinated	A child between 12–23 months old who received: BCG, DtwPHibHepB-3, OPV-3, Yellow Fever and Measles vaccines at the time of survey
Fully vaccinated before 12 months of life	A child between 12–23 months old who received all the above vaccines by the age of 12 months (it is evaluated only by vaccination card)
Partially vaccinated	A child between 12–23 months old who missed at least one dose of the above vaccines
Unvaccinated	A child between 12–23 months old who does not receive any dose of the above vaccines

*BCG* bacille calmette guérin, *OPV* oral polio vaccine, *DTwPHibHepB* diphtheria and tetanus toxoid with whole cell pertussis, *Haemophilus Influenzae* type B and hepatitis B vaccines

Relative frequencies and other descriptive statistics were calculated to evaluate the immunization coverage and the association of the vaccination uptake (by card plus parents’ recall) with the independent variables. Statistical inference [i.e., 95 % confidence intervals (95 % CI) and p-values] was performed taking into account the two-stage sampling design [13]. Finally, to calculate adjusted odds ratios (AOR) of factors independently associated with incomplete immunization coverage (by card plus parents’ recall), a multiple multilevel logistic regression model was performed. Regarding the selection of this multiple model, initially we planned, following the indication by Hosmer and Lemeshow [27] to select the variables to be included in the final model based on the results of the univariate analysis (i.e., including only those with a log-likelihood ratio test <0.20) and then to further exclude among those initially included in the multiple model that, at each step, had the highest log-likelihood ratio test p-value ≥0.20 until only variables with a p-value <0.20 were remaining. However, this approach actually was not possible because the multiple model based on the characteristics selected at the univariate analysis (i.e., those with *p* < 0.20) did not converge. We then decided to select the characteristics to be included on the basis of “intermediate” multiple logistic models performed for each section of the survey questionnaire (i.e., children, parents, household characteristics and parents’ perceptions, knowledge and attitudes). The variables were further selected by a subsequent backward selection that at each step excluded that variable with the highest p-value >0.20 (from log-likelihood ratio test) until only variables with a p-value <0.20 remained. All analyses were performed using STATA software (version 12.0).

### Ethics approval

Ethical approval was obtained from the *“Comité National d’éthique de la Recherche pour la Santé Humain du Cameroun”* and Dschang Health Authority. Data collection was conducted confidentially and after obtaining verbal parents’ informed consent. Partially vaccinated children were notified to Dschang Health Authority in order to complete the recommended vaccinations.

## Results

A total of 3248 households were visited, and 5 additional clusters were sampled in order to achieve the expected sample size. A total of 502 parents of children aged 12-to-23 months recruited in 65 clusters were interviewed with a response rate of 100 %. The average number of children recruited per cluster was 7.72 (range 2–9), and the average number of households visited per cluster before finding up to 9 eligible children was 49.96 (range 13–99).

### Study population characteristics

The children’ mean age was 19.6 months; of them, 50.6 % were male, and the vast majority (97.6 %) were born at health facilities. The most represented parents’ age group was 25–29 years for mothers (40.7 %) and ≥30 years for fathers (70.7 %). With regard to the households, 87.2 % were two parents-family, 51.4 % of Catholic religion; 41.2 % had more than three children <5-years old, and 23.7 % were distant >8 km from the nearest vaccination centre. Only 26.6 % of the parents had secondary education. The principal fathers’ work activity was farmer (27.1 %), while many of the mothers were not working/unemployed (35.8 %); 53.6 % of households belonged to the economic poorest tertile. The majority of the mothers (64.5 %) reported ≥3 ANCs during the last pregnancy, but only 29.1 % reported at least one postnatal visit. Respectively 85.4 % and 50.2 % of the parents showed positive attitude and good knowledge about vaccination, 78.5 % declared to receive regularly information on vaccination, and 80.5 % reported a good opinion on vaccination services of the Dschang health district.

### Immunization coverage by card only

Vaccination card was available for 252 (50.2 %) children. As shown in Table 3, 84.5 % (95 % CI 79.5–89.5) of the children were fully immunized, but only 73.4 % (95 % CI 67.6–78.4) within one year of life, while 15.5 % (95 % CI 11.5–20) were partially immunized. The most frequently administered vaccines were BCG (98.8 %) and DTwPHibHepB-1 (Diphtheria and tetanus toxoid with whole cell pertussis, *Haemophilus Influenzae* type B and Hepatitis B vaccines) (98 %), while measles and yellow fever were the least taken vaccines (90 %). OPV-3 was received by 91.6 % of children. DTwPHibHepB-1/3 and OPV-1/3 dropout rates were respectively 5.3 and 4.2 %, while the dropout rate from BCG to measles and yellow fever vaccination was 8.9 %.

**Table 3:** Immunization coverage by card and by card plus parents’ recall in children aged 12–23 months, Dschang (Cameroon), Nov. 2013

Vaccine	Card plus parents’ recall (*n* = 502), % (95 % CI)	Card only (*n* = 252), % (95 % CI)
BCG	99.8 (98.8–99.9)	98.8 (96.5–99.5)
DTwPHibHepB – 1	98.8 (97.4–99.4)	98 (95.4–99.1)
DTwPHibHepB – 2	98.2 (96.6–99)	97.6 (94.9–98.9)
DTwPHibHepB – 3	94.8 (92.5–96.4)	92.8 (89–95.4)
OPV – 0	97.6 (95.8–98.6)	93.6 (89.9–96)
OPV – 1	98.4 (96.8–99.1)	95.6 (92.3–97.5)
OPV – 2	97.8 (96.1–98.7)	95.2 (91.8–97.2)
OPV – 3	95 (92.7–96.6)	91.6 (87.6–94.4)
Measles	91.2 (88.4–93.4)	90 (85.7–93.1)
Yellow Fever	91.2 (88.4–93.4)	90 (85.7–93.1)
Unvaccinated^a^	0 (0–0.7)	0 (0–1.4)
Partially vaccinated^a^	14.1 (10.7–17.6)	15.5 (10.5–20.5)
Fully vaccinated^a^	85.9 (82.4–89.3)	84.5 (79.5–89.5)
Fully vaccinated ≤1 year^a^		73.4 (67.6–78.4)

*BCG* bacille calmette guérin, *OPV* oral polio vaccine, *DTwPHibHepB* diphtheria and tetanus toxoid with whole cell pertussis, *Haemophilus Influenzae* type B and hepatitis B vaccines

^a^For children vaccination status definition, see Table 2

### Immunization coverage by card plus parents’ recall

As shown in Table 3, based on data from vaccination card plus parents’ recall, 85.9 % (95 % CI 82.4-89.3) of the children were fully immunized, and 14.1 % (95 % CI 10.7-17.6) were partially immunized; unvaccinated children were not found. Among partially immunized children, the main reported mothers’ reason for not being able to vaccinate their children was “to be very busy” (32.3 %, *n* = 22). Among the nine vaccines, BCG and DTwPHibHepB-1 were the most frequently administered (99.8 % and 98.8 % respectively), while measles and yellow fever were the least taken vaccines (91.2 %). OPV-3 was received by 95 % of children. DTwPHibHepB-1/3 and OPV-1/3 dropout rates were respectively 4 % and 3.4 %, while the dropout rate from BCG to measles and yellow fever vaccination was 8.6 %.

### Factors associated with incomplete immunization coverage: univariate analysis

Table 4 shows the univariate association between the study variables and the children immunization status, assessed by card plus parents’ recall. Children born at health facilities had a higher immunization coverage rate compared with those born at home (*p* < 0.01), as well as children who were the 1^st^-2^nd^ born in the family compared with those being the 3^rd^ or later born (*p* < 0.01). Children from low-educated and younger parents were less immunized (*p* < 0.01), as well as children from poorest household (*p* = 0.01) and from single-parent family (*p* < 0.01). The distance of the household from the vaccination centre was found associated with the immunization status (*p* < 0.01). Moreover, children of mothers who had ≥3 ANCs and at least one postnatal visit had better immunization coverage (*p* < 0.01 and *p* = 0.03 respectively). Finally, children of parents who had good knowledge and positive attitude towards immunization, who received regularly information on vaccinations and who had a good opinion of Dschang immunization services, were more likely to be fully vaccinated (*p* < 0.01).

**Table 4:** Socio-demographic characteristics^a^ associated with the children immunization status (by card plus parents’ recall) at the univariate analysis (row Chi-square), Dschang (Cameroon), Nov. 2013

			Partially immunized		Fully immunized		Total	*p*-value
			N	%	N	%	N	
Children characteristics	Birth order	≤2nd	6	1.8	325	98.2	331	<0.01
		≥3rd	65	38	106	62	171	
	Place of birth	Hospital/Health Facilities	59	12.1	430	87.9	489	<0.01
		Home	12	92.3	1	7.7	13	
Parents’ characteristics	Mother’s age group	≤24	35	34	68	66	103	<0.01
		25–29	15	7.3	189	92.7	204	
		≥30	21	10.8	174	89.2	195	
	Father’s age group	≤24	15	65.2	8	34.8	23	<0.01
		25–29	20	16.1	104	83.9	124	
		≥30	36	10.1	319	89.9	355	
	Mother’s education	Primary education	49	18.1	221	81.9	270	<0.01
		Secondary education	6	4.8	119	95.2	125	
		High school/University	16	14.9	91	85.1	107	
	Father’s education	Primary education	45	21.5	164	78.5	209	<0.01
		Secondary education	8	5.6	134	94.4	142	
		High school/University	18	12	132	88	150	
	Mother’s occupation	Unemployed	36	20	144	80	180	0.03
		Trader	12	10.4	103	89.6	115	
		Craftsman	4	11.8	30	88.2	34	
		Farmer	6	6.4	88	93.6	94	
		Official	6	13	40	87	46	
		Professionals	7	21.2	26	78.8	33	
	Parent’s Religion	Catholic	34	13.2	224	86.8	258	0.03
		Protestant	3	5.3	54	94.7	57	
		Muslims	13	14.6	76	85.4	89	
		Atheists	21	21.4	77	78.6	98	
	Number of pre-natal visits	≤2	64	36.2	113	63.8	177	<0.01
		≥3	7	2.2	317	97.8	324	
	Number of post-natal visits	None	58	16.3	297	83.7	355	0.01
		≥1	13	8.9	133	91.1	146	
Household characteristics	Type of family	2-parents family	38	8.7	400	91.3	438	<0.01
		Single-parent family	33	51.6	31	48.4	64	
	Socio-economic tertiles	Poorest	48	17.8	221	82.2	269	0.01
		Middle	9	6.9	122	93.1	131	
		Richest	14	13.7	88	86.3	102	
	Distance to vaccination centre	≤7 Km	36	9.4	347	90.6	383	<0.01
		≥8 Km	35	29.4	84	70.6	119	
	N° of children <5 years old in the family	≤2	11	4	265	96	276	<0.01
		≥3	45	21.7	162	78.3	207	
	Family-living time in Dschang	≤2 years	7	16.3	36	83.7	43	<0.01
		3–6 years	32	32	68	68	100	
		≥7	32	8.9	327	91.1	359	
Parents’ perceptions, knowledge and attitudes	Vaccination knowledge score	Good	2	0.8	250	99.2	252	<0.01
		Poor	69	27.6	181	72.4	250	
	Attitude towards immunization	Positive	24	5.6	405	94.4	429	<0.01
		Negative	47	64.4	26	35.6	73	
	Parents receive regularly information on vaccinations	Present	10	2.5	384	97.5	394	<0.01
		Absent	61	57	46	43	107	
	Opinion of Dschang immunization services	Good	37	9.2	367	90.8	404	<0.01
		Bad	21	26.6	58	73.4	79	

^a^Only significant association included [children sex (*p* = 0.43), possessing immunization card (*p* = 0.41), father’s occupation (*p* = 0.07), and family ethnic group (*p* = 0.94) were not associated with immunization status at the univariate analysis]

### Factors associated with incomplete immunization coverage: multilevel logistic regression analysis

Table 5 shows the estimated AORs obtained by the multilevel logistic regression analysis. In the final model, the characteristics significantly associated with incomplete children immunization status were: having the vaccination card (AOR 7.89; 95 % CI 1.08–57.3; *p* = 0.04), being the 3^rd^ or later born child in the family (AOR 425.4; 95 % CI 9.62–18,808; *p* < 0.01), having a mother ≤24 years-old (AOR 49.55; 95 % CI 1.59–1544; *p* = 0.03), having a mother who attended ≤2 ANCs (AOR 8.25; 95 % CI 1.07–63.7; *p* = 0.04), having parents with negative attitudes towards vaccination (AOR 20.20; 95 % CI 1.46–278.9; *p* = 0.03) and who do not regularly receive information about immunization (AOR 28.07; 95 % CI 2.26–348; *p* < 0.01). Finally, the association with longer distance (≥8 Km) from the vaccination centers was marginally significant (AOR 6.44; 95 % CI 1.00–41.4; *p* = 0.05).

**Table 5:** Adjusted odds ratios of incomplete immunization (by card plus parents’ recall) in children aged 12–23 months, Dschang (Cameroon), Nov. 2013

Independent variable		AOR^a^	95 % CI		p	AOR^b^	95 % CI		*p*-value
Children characteristics	Immunization card (yes vs. no)	1.59	0.8	3.15	0.18	7.89	1.08	57.37	0.04
	Birth order (≥3rd vs. ≤2nd)	38.27	14.6	100.36	<0.01	425.47	9.62	18808.1	<0.01
	Place of birth (home vs. health facilities)	73.62	5.57	972.42	<0.01	NI			
Parents’ characteristics	Mother’s age group (≤24 vs. ≥25)	10.5	4.3	25.59	<0.01	49.55	1.59	1544.5	0.03
	Father’s age group (≤24 vs. ≥25)	NI				NI			
	Mother’s education (secondary vs. primary)	3.75	1.14	12.33	0.03	NI			
	Mother’s education (High school/University vs. primary)	5.86	1.58	21.83	<0.01				
	Father’s education (secondary vs. primary)	NI				NI			
	Father’s education (High school/University vs. primary)								
	Mother’s occupation (Unemployed vs. Farmer )	NI				NI			
	Mother’s occupation (Trader vs. Farmer)								
	Mother’s occupation (Craftsman vs. Farmer)								
	Mother’s occupation (Official vs. Farmer)								
	Mother’s occupation (Professionals vs. farmer)								
	Father’s occupation (Unemployed vs. Farmer)	NI				NI			
	Father’s occupation (Trader vs. Farmer)								
	Father’s occupation (Craftsman vs Farmer)								
	Father’s occupation (Official vs. Farmer)								
	Father’s occupation (Professionals vs. Farmer)								
	Parent’s Religion (Catholic vs. Protestant)	NI				NI			
	Parent’s Religion (Atheists vs. Protestant)								
	Parent’s Religion (Muslims vs. Protestant)								
	Number of pre-natal visits (≤2 vs. ≥3)	40.98	14.4	116.63	<0.01	8.25	1.07	63.75	0.04
	Number of post-natal visits (0 vs. ≥1)	NI				NI			
Household characteristics	Family ethnic group (local/Bamiléké vs. other)	NI				NI			
	Type of family (Single-parent vs. 2-partens)	13.96	5.72	34.08	<0.01	3.87	0.53	28.48	0.18
	Socio-economic tertiles (Poorest vs. Middle)	NI				NI			
	Socio-economic tertiles (Poorest vs. Richest)								
	Distance to vaccination centre (≥8 Km vs. ≤7)	4.96	2.18	11.29	<0.01	6.44	1	41.39	0.05
	N° of <5-ywears old in the family (≥3 vs. ≤2)	12.02	4.83	29.9	<0.01	NI			
	Family-living time in Dschang (≤2 vs. 3–6 years)	6.24	2.36	16.5	<0.01	NI			
	Family-living time in Dschang (≤2 vs. ≥7 years)	2.63	0.99	6.97	0.05				
Parents’ perceptions, knowledge and attitudes	Vaccination knowledge score (poor vs. good)	9.12	1.72	48.35	<0.01	8.88	0.57	137.15	0.12
	Attitude towards immunization (negative vs. positive)	8.27	2.95	23.17	<0.01	20.2	1.46	278.91	0.03
	Opinion of Dschang immunization services (bad vs. good)	9.16	3.13	26.77	<0.01	4.03	0.7	23.35	0.12
	Parents regularly informed on vaccinations (absent vs. present)	18.35	5.94	56.73	<0.01	28.07	2.26	348.13	<0.01

*COR* crude odds ratio, *AOR* ^*a*^ adjusted odds ratio model 1, *AOR* ^*b*^ adjusted odds ratio final model, *NI* not included in the final multilevel multiple logistic regression model

## Discussion

We assessed the routine vaccination coverage and risk factors for incomplete vaccination among children aged 12–23 months in the Dschang district, West Region, Cameroon. The survey was performed during a polio outbreak occurred in the same region of the country [5, 8]. Thus, OPV-3 coverage data reported from our study may represent an indicator for the risk assessment of WPV spread in the study area. Vaccination coverage was assessed by card only and by card plus parents’ recall. Immunization cards were available for 50.2 % of the children. As shown in Table 6, the vaccination coverage observed in this survey was higher than that reported for the whole Cameroon by WHO [28], and by the 2011 Cameroonian DHS for the whole country and the West Region [4].

**Table 6:** Vaccines coverage (by card plus parents’ recall) compared to latest data for Cameroon from WHO and Demographic Health Survey (DHS)

Vaccine	Present study (*n* = 502)	WHO (2013) [26]	DHS (2011) [4]	
			Cameroon (n=2265)	West Region, Cameroon (n=272)
BCG	99.8	82	87.1	95.9
DTwPHibHepB – 1	98.8	95	85.5	94.4
DTwPHibHepB – 2	98.2	89	78.3	87
DTwPHibHepB – 3	94.8	89	68.4	75.5
OPV – 0	97.6	n.a	71.7	84.4
OPV – 1	98.4	n.a	93.3	92.5
OPV – 2	97.8	n.a	85.5	87.5
OPV – 3	95	88	69.8	76.6
Measles	91.2	83	70.6	79.8
Yellow Fever	91.2	83	69.3	77.7
Not vaccinated	0	n.a.	4.5	2.3
Partially vaccinated	14.1	n.a.	49.8	40.7
Fully vaccinated	85.9	n.a.	50.2	59.3
Fully vaccinated ≤12 months ^a^	73.4	n.a.	45.1	n.a.

*BCG* bacille calmette guérin, *OPV* oral polio vaccine, *DTwPHibHepB* diphtheria and tetanus toxoid with whole cell pertussis, *Haemophilus Influenzae* type B and hepatitis B vaccines, *n.a.* not available

^a^vaccine coverage assessed by card only

According to card plus parents’ recall, 85.9 % of the children of our survey were fully vaccinated. Vaccination coverage reported by immunization surveys in rural African areas ranged from rates as low as 26 % in Burkina Faso [18] to 88 % in Ghana [29]. Moreover, in our survey, there was no unvaccinated child, confirming the low rate (2.3 %) reported by the 2011 Cameroonian DHS for the West Region [4]. For comparison, unvaccinated children ranged from 6.5 % [24] to 24 % [20] in surveys conducted in Ethiopia. According to immunization card plus parents’ recall, we observed 14.1 % of partially vaccinated children (15.5 % by card only), a lower proportion than that reported by surveys conducted in Mozambique (28.2 %) [22] and Ethiopia (20.3 %) [24]. Although our findings are indicative of a rather high adherence to vaccination schedule in term of completeness, 26.6 % of children did not complete the recommended doses within one year of life; no comparable data were found in the scientific literature. DTwPHibHepB-3 coverage in our survey was 94.8 % by card plus parents’ recall (92.8 % by card only), which is higher than what reported by other surveys performed in rural Ethiopia (47.9 %) [20], Nigeria (80.8 %) [16], and Kenya (90 %) [19]. OPV-3 immunization coverage was >90 %, based on both card plus parents’ recall and card only, indicating that 12–23 months old children living in the Dschang district had a good protection level against the risk of WPV spread related to the ongoing polio outbreak in the West Region of Cameroon [5, 8]. Other surveys performed in Africa reported OPV-3 coverage of 91.8 % in Kenya [19], 80.8 % in Nigeria [16], 86.3 % and 54.3 % in Southern [24] and Central Ethiopia [20], respectively.

Limited differences were found in vaccination coverage when assessed by card plus parents’ recall or by card only. An expected tendency of highest vaccination coverage values when assessed by card plus parents’ recall was observed, in accordance with WHO and the international literature [10, 20]. The biggest difference was related to OPV-0 (+4 %), though it was lower than that reported in a study from Ethiopia [20], where differences ranged from 12 % to 34 %. In our study, as also reported by the 2011 Cameroonian DHS [4] and by another survey from Kenya [30], OPV-0 coverage (by card plus parents’ recall and by card only) was lower than BCG and OPV-1 coverage. The reason of this finding is undefined; OPV-0 could be underreported in the card by health care staff and/or less recalled by parents when compared to BCG vaccine, that is intradermally administered at birth, and to OPV-1 that is administered with DTwPHibHep-1 at 6th weeks of life. No difference was observed between measles and yellow fever vaccination coverage when assessed by card plus parents’ recall or by card only (Table 3), consistently with data reported by the 2011 Cameroonian DHS [4] and by a study from Sierra Leone [31]. This finding suggests that these two vaccines are correctly administered during the same vaccination session. The vaccination dropout rates observed are acceptable, according to the WHO standard (cut-off < 10 %) [26], and lower than those reported by studies from Ethiopia [23, 24].

Several characteristics were associated with immunization status in our study, but only six of them were statistically significant at the final multilevel regression model (Table 5). Birth order was statistically associated with fully immunization, similarly to what reported by studies from Kenya [19] and India [32], but in contrast with a survey from Ethiopia [20]. This finding could be related to a reduced mothers’ attention along with growing number of children, due to an increase of duties, as confirmed by mothers’ interview (the main reported reason for not being able to vaccinate their child was “to be very busy”). Being born at the hospital/health facility was linked to complete vaccination status in studies from different African countries [19, 20, 22], while in our survey it was associated in a restricted multilevel model including only the children characteristics, but the association was no longer statistically significant when adjusting for all other factors. Perhaps, the effect of place of birth was explained in the model by prenatal visits and/or access to health services, of whom it may be considered a proxy. A lower number of mothers’ ANCs and the younger mothers’ age were associated with incomplete vaccination status, as in other studies from African countries [7, 16, 20, 29, 33]. It is possible that reduced contacts with health facilities during pregnancy, especially among younger mothers, could cause poorer information toward immunization. A longer distance (≥8 Km) from vaccination sites was marginally significant (*p* = 0.05) in the final model. The accessibility to vaccination sites was associated to immunization status in studies from rural Mozambique [21] and Nigeria [16], while no distance related difference was found in a study from Burkina Faso [18]. Mothers’ education was associated in the first multilevel model, including only parents’ characteristics, but no more when all factors were included in the model; this covariate was found associated with complete vaccination in several studies from African countries [7, 16, 18, 29].

A high vaccination knowledge score, positive attitudes toward vaccination, good perception of immunization services, and the exposure to information on vaccinations, were all associated with complete vaccination status. As in other studies from Nigeria [16], Niger [33], and India [32], despite a parents’ poor vaccination knowledge score in 50 % of the cases, the majority of interviewed parents (84.5 %) had a positive attitude toward immunization. Overall, only parents’ positive attitude and regular information received remained significantly associated with complete vaccination in the final multilevel model. These two factors were reported as determinants of complete immunization status in a multilevel analysis involving 24 sub-Saharan African countries [7]. Concerning these aspects, it is noteworthy that the father of the index case of the polio outbreak recorded in the West Region in Cameroon (October 2013) was a political leader and pastor that, despite its good education level and high economic status, was strongly opposed to vaccination [8], and his children had never been vaccinated.

Other demographic factors linked to vaccination status identified in studies conducted in several African countries were found either not statistically associated or associated only at the univariate analysis, or were just not considered in our study. Among these factors, parents’ socio-economic status and number of children in the family were not significant in the final multilevel model, while they were significantly associated in other African studies [18, 28], including one from Cameroon [34]; birth season and residence area, significantly associated in a study from Burkina Faso [18], were not considered in our survey. Surprisingly, in contrast with the results of surveys conducted in African countries [16, 18, 22] and India [32], possessing the vaccination card was associated with incomplete vaccination. This, as suggested by WHO [10], might be due to parents over-reporting of vaccine doses for complacency, without the possibility to check the information. This aspect is controversial and may represent a limit of our study. In fact, a recent systematic review on the validity of vaccination card and parents’ recall to estimate vaccination coverage [35] suggests that parents’ recall information should be cautiously interpreted because it might be not reliable. This issue is not mentioned in WHO EPI-coverage survey guidelines [10] and has not been addressed in the fields’ immunization surveys [16–24]; thus, methodological studies on this aspect are needed.

Another limit is that our survey was conducted in a district of a region affected by a polio-outbreak situation, and two SIAs have been realized before and during our study. This situation could introduce biases in the OPV coverage evaluation for different reasons: firstly, vaccine doses may have been administered from other than Dschang vaccination facilities; secondly, parents could incorrectly report vaccine doses as administered during SIAs or routine immunization and vice-versa. Thus, the contribution of routine immunization activities and SIAs to the OPV coverage rate could not be completely distinguished. However, coverage rates for OPV-3 were similar to DTwPHibHepB-3, as well as all observed coverage for the different vaccines administered during the same routine immunization session. Furthermore, even if the parents’ tendency to over-report doses of vaccines [10, 20] may introduce biases, vaccines coverage rates assessed by card plus parents’ recall and by card only were similar in our study.

A further study limitation concerns the procedure used to determine households’ economic status. In fact, our specific algorithm was based only on the type of family (two or single-parent) and parents’ occupation, while housing conditions and economic activities of other household members were not considered. The sampling procedure was also susceptible of selection bias and the study did not include qualitative methods to address this questions. Moreover, qualitative methods, such as in-depth interviews, were not used to investigate the reasons for partial or delayed vaccination. Planning qualitative studies may be a useful complement to quantitative surveys in better understanding obstacles and possible solutions to increase vaccination coverage.

To our knowledge, despite the above mentioned limitations, this is one of the few studies using a multilevel logistic regression analysis to identify risk factors for incomplete immunization status and the intracluster correlation coefficient for the sampling size calculation. Our study contributes to the identification of factors related to the children immunization status at a district level in Cameroon, where similar studies are lacking. Moreover, we reported the vaccination coverage in children <12 months of age, that is not mentioned in surveys performed in similar African contexts, and we provided an important risk indicator of WPV spread during the ongoing outbreak situation.

## Conclusion

In conclusion, our study revealed high vaccination coverage in the Dschang district (West Region, Cameroon), and a good protection level against the risk of WPV spread. However, although none of the children was completely unvaccinated, almost 1 out of 7 children was partially vaccinated, and almost 1 out of 4 children did not complete the recommended vaccinations within 12 months of life. Younger parents and families living far away from vaccination centres should be targeted with appropriate immunization promotion strategies, and the improvement of prenatal care and delivery services should be addressed in order to increase immunization coverage and proper (timely) vaccines administration. Information and attitude towards immunization should be strengthened with adequate vaccination education programmes, in order to favour this evidence-based intervention [36, 37].
